# Mechanisms and Signals for the Nuclear Import of Proteins

**DOI:** 10.2174/138920209789503941

**Published:** 2009-12

**Authors:** Natália Freitas, Celso Cunha

**Affiliations:** Unidade de Biologia Molecular, Centro de Malária e outras Doenças Tropicais, Instituto de Higiene e Medicina Tropical, Universidade Nova de Lisboa, Rua da Junqueira, 96 1349-008 Lisboa, Portugal

**Keywords:** Nuclear pore complex, nuclear localization signal, importin, nuclear transport.

## Abstract

In eukaryotes, the nuclear membrane provides a physical barrier to the passive diffusion of macromolecules from and into the cytoplasm. Nucleocytoplasmic traffic occurs through highly specialized structures known as nuclear pores, and involves the participation of a special class of transport proteins. Active transport across the nuclear pores is an energy-dependent process that relies on the activity of Ran-GTPases both in the nuclear and cytoplasmic compartments.

Nuclear import of proteins is an essential step in regulating gene expression and the replication cycle of several viruses. In this review, the key mechanisms, pathways, and models underlying the transport of proteins across nuclear pores are analysed.

## NUCLEAR PORE COMPLEX

**Fig. (1):**
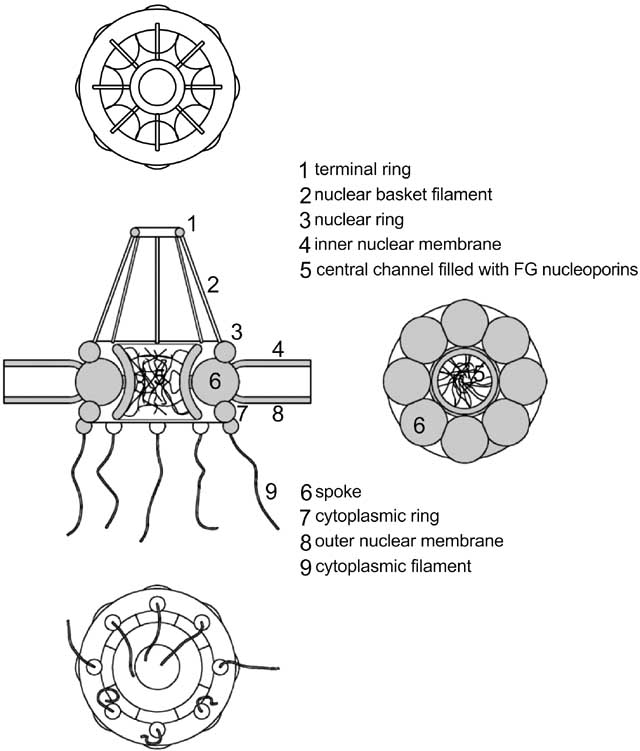
Schematic view of the nuclear pore complex. The crosssection of the nuclear ring, central structure, and cytoplasmic ring are displayed on the top, middle, and bottom of the figure, respectively.

In eukaryotic cells the nucleus is physically separated from the cytoplasm by a double membrane structure, the nuclear envelope (NE). The NE is crossed by multiple supramolecular structures specialized in mediating the bidirectional traffic of molecules between the nucleus and the cytoplasm. These structures are designated the nuclear pore complex (NPC). The number of NPCs per cell is variable between species and is dependent on cell size and transcriptional activity. It is generally estimated that the rate of translocation through NPCs may achieve 1000 molecules per second. The nucleus of yeast cells usually contains about 200 NPCs while human cells and mature *Xenopus* oocytes may contain 5x10^3^ – 5x10^7^ NPCs per nucleus [1]. Electron microscopy observations have shown that, in most cases, the three dimensional structure of the NPC is conserved even in evolutionary distant species [2]. NPCs display a cylindrical tripartite structure which is about 90 nm in length and 50 nm wide (Fig. **1**). The central structure of the NPC is anchored between the inner and outer layers of the NE. This central structure consists of eight subunits that constitute the nuclear and cytoplasmic rings [3]. Eight protein filaments of 50 nm in length originate from the cytoplasmic ring towards the cytoplasm. From the nuclear ring also originate eight filaments. The filaments in the nuclear side, however, are 75 nm in length and converge to a ring-like structure named the nuclear basket [3]. The central structure of the NPC contains a central channel [3]. This channel is about 30 nm in diameter and allows the transport, by passive diffusion, of ions and small molecules, including proteins with a molecular mass up to 40 KDa. The traffic of larger molecules through the NPCs requires, however, the involvement of energy dependent active transport mechanisms [4]. 

Early estimates of the number of proteins in NPCs seemed to indicate that these complex structures may contain up to 100 different proteins. More recently, proteomic approaches have been used to analyse the protein composition of NPCs, both in yeast and vertebrates. Surprisingly these experiments revealed that NPCs are constituted by only 30 different proteins which are generally designated as nucleoporins [5, 6]. About one third of the identified nucleoporins contain a characteristic structural motif constituted by multiple repeats of phenylalanine and glycine residues (FG motifs) [5]. The majority of nucleoporins are symmetrically distributed relative to the middle plan of the NE [6]. Based on the relative abundance and molecular mass of each nucleoporin it was possible to estimate that the molecular mass of the NPC may range between 44 and 60 MDa in yeast and vertebrates, respectively [5, 6]. These values are distinct from those initially obtained by transmission-scanning microscopy which ascribed molecular masses of 60 MDa for the yeast NPC and 125 MDa for the vertebrate NPC [7, 8].

## PROTEIN TRANSPORT RECEPTORS

For the majority of macromolecules, the nucleocytoplasmic transport through the NPCs is an energy-dependent process mediated by soluble transport receptors that generally belong to a family of proteins designated β-karyopherins [9]. β-karyopherins mediate both the import and export of all proteins displaying dimensions over the size exclusion limit for simple diffusion through the NPCs [9]. Besides this, β-karyopherins also mediate the transport of non-coding cellular RNAs [10]. The formation of import or export complexes is dependent on the interaction of β-karyopherins with small peptide motifs present in protein cargos. These motifs are generally called nuclear localization signals (NLS) or nuclear export signals (NES). The molecular mechanisms underlying RNA transport are usually more complex since they include the additional participation of adaptor proteins that interact with the transport receptor and the RNA [10]. 

**Fig. (2):**
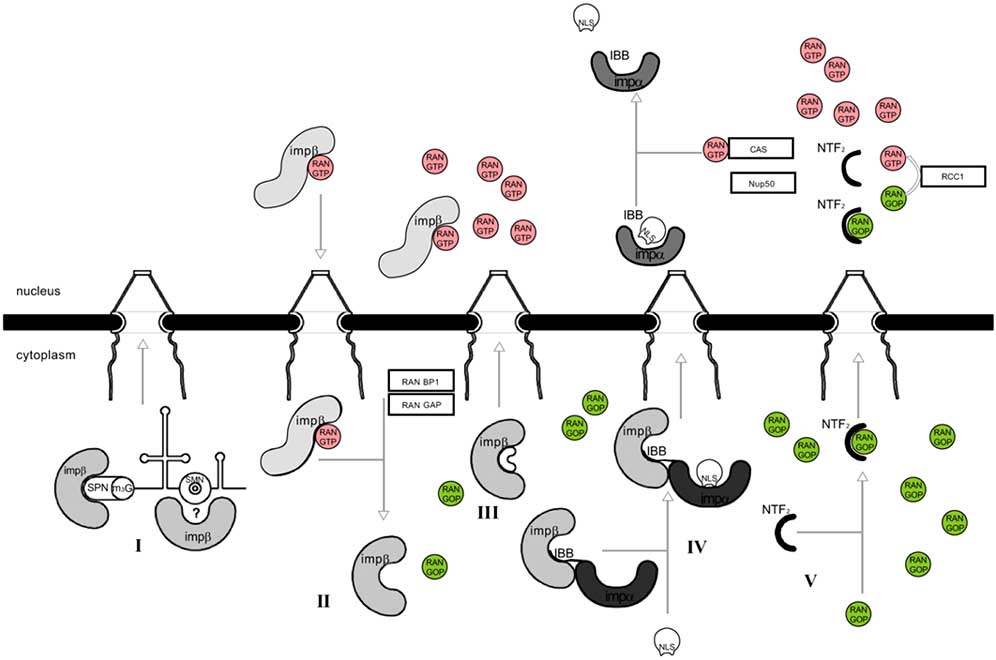
Schematic representation of protein nuclear import pathways. **I-** Import of UsnRNPs is mediated by importin-β which associates with the SMN complex coupled to UsnRNAs; **II-** The RanGTP gradient regulates the shuttling of importin-β between the nucleus and the cytoplasm; **III-** The direct interaction of importin-β with cargos promotes translocation through nuclear pores of several proteins; **IV-** Importin-α recognizes the NLS in the cargos and interacts with importin-β through the IBB domain to promote nuclear import; **V-** The nuclear import of Ran-GDP is mediated by the nuclear transport factor NTF2.

β-karyopherins are acidic proteins with molecular masses ranging from 90 to 145 KDa. In eukaryotes, over 20 β-karyopherins were identified of which 10 were shown to directly participate in the nuclear import of proteins [9]. β-karyopherins are characterized by the ability to directly interact with both the Ran GTPase and the FG domains of nucleoporins [11]. Ran GTPase is a monomeric protein of 24 KDa that belongs to the Ras superfamily. Similar to other GTPases, Ran can be found in two distinct forms: GTP-bound and GDP-bound. The two forms of Ran are asymmetrically distributed between the nucleus and the cytoplasm. Ran-GTP localizes predominantly in the nucleus while Ran-GDP is observed mainly in the cytoplasm [12]. The asymmetric distribution of the two forms of Ran is maintained by specific regulatory proteins localized in the nuclear and cytoplasmic compartments. Usually, as in the case of other members of the Ras superfamily, the intrinsic GTPase activity of Ran, promoting the hydrolysis of GTP into GDP, is slow. However, this activity can be accelerated by two cytoplasmic proteins: Ran-GAP and RanBP1 (Fig. **2-II**) [12]. The reverse reaction, conversion of Ran-GDP into Ran-GTP is stimulated by the nuclear protein RCC1. RCC1 binds to Ran-GDP promoting GDP dissociation and subsequent binding of GTP in the active centre of Ran (Fig. **2-V**). The combined action of Ran regulatory proteins creates and maintains a Ran-GTP gradient across the NE. This gradient is a key element in establishing the direction of nucleocytoplasmic transport. In fact, the interaction of β-karyopherins with the respective cargo substrates is regulated by the Ran-GTP gradient [13, 14]. 

Along with the action of Ran regulatory proteins the Ran-GTP gradient is also maintained by the nuclear import of Ran-GDP. The nuclear transport of Ran-GDP is regulated by the NFT2 import factor [15, 16]. NTF2 is a conserved 15 KDa protein that specifically binds to the GDP-bound form of Ran [15]. The nuclear import of the complex Ran-GDP-NFT2 is not energy dependent. Once in the nucleus, this complex dissociates after conversion of Ran-GDP into Ran-GTP by the action of the nuclear protein RCC1 (Fig. **2-V**) [15]. 

## NUCLEAR TRANSPORT MODELS

In spite of the progress made towards the understanding of the architecture and protein composition of the NPC, the mechanisms by which this specialized protein complex acts as a selective barrier still remains elusive. Nevertheless, it has been shown that the interaction of transport receptors with the FG motifs of nucleoporins is an essential step for translocation through NPCs [17, 18]. According to this view, it was also demonstrated that removal of the FG binding motifs of importin-β results in the total abolishment of the import pathways mediated by this transport receptor [19, 20]. 

Based, at least partially, on experimental evidences, a number of models were proposed in an attempt to explain the mechanisms of transport through NPCs.

In 2000, Rout *et al*. proposed a model where the presence of numerous nucleoporin filaments on both sides of the central channel of the NPC is thought to confer resistance to passive diffusion for large macromolecules [6]. In addition, the authors postulated that the complex of macromolecules and the respective transport receptors may become prone to reside for increased periods of time in the vicinity of the central channel of the NPC, and thus augmenting the probability to cross it. The vectorial transport through NPCs is then established by the asymmetric distribution of Ran-GTP and some nucleoporins. In summary, this model supports the existence of a physical barrier constituted by the nucleoporin filaments on both sides of the central channel of the NPC. This barrier hampers the transport of proteins and RNAs that are not linked to the respective transport receptors [21]. 

One year later, Ribbeck and Gorlich proposed an alternative selective phase model. According to this model, the central channel of the NPC contains FG- rich nucleoporins that interact to form a hydrophobic network [22]. This network acts as a mesh to exclusively allow the transport of small molecules. For molecules of larger size the transport is allowed only when they are linked to transport receptors. These cargo-receptors complexes may then recognize the FG motifs in nucleoporins and subsequently cause the local disruption of the network. In support of this model, Frey *et al*. showed that the FG motifs of the yeast nucleoporin Nsp1p may form *in vitro*, hydrogel-like structures [23]. These structures are elastic and mechanically stable. The formation of this type of hydrogels is dependent on the presence of FG motifs. Moreover, in yeasts the substitution of phenilalanine by serine residues in the FG motifs of Nsp1p does not allow the formation of hydrogels and was shown to be lethal [23].

The observations that the binding affinity of importin-β to the FG motifs of nucleoporins progressively increases along the inner side of the NPC, and that importin-β contains more than one interaction domain with FG nucleoporins, led Ben-Efraim and Gerace to propose a third model for nuclear transport [24]. According to this model, the movement of the cargo-receptor complex is favoured in the cytoplasm-nucleus direction due to the increasing affinity of importin-β to the nucleoporins localized in the nuclear side of the NPC. However, the mechanism by which the import complexes are released from the first nucleoporin binding site in order to further bind to the following nucleoporins, still needs to be clarified. It is possible that the simultaneous interaction of import complexes with two nucleoporins may help promoting the dissociation of the complex with the nucleoporin displaying less affinity. This dissociation would simultaneously promote the subsequent tight association with the next nucleoporin. This affinity gradient model anticipates that the export complexes move across the NPC in a way similar to that above described for the importin-β mediated import pathways. This model is based on the asymmetric distribution of some of the NPC components, and suggests that the transport direction is imposed by the NPC itself. However, a number of observations do not seem to be in accordance with this model. First, the majority of FG nucleoporins is symmetrically distributed in the NPCs. Second, the asymmetrically distributed nucleoporins were found to be usually dispensable for the majority of nuclear transport pathways [25]. Finally, the observation that the direction of nuclear transport may be inverted in the presence of high concentrations of Ran-GTP in the cytoplasm indicates that the NPC does not directly rule the direction of the nucleocytoplasmic traffic [26].

## NUCLEAR LOCALIZATION SIGNALS

The first evidence that the nuclear transport of proteins is mediated by specific peptide signals came from the observation that the proteolytic hydrolysis of the C-terminal aminoacids of nucleoplasmin hampers its migration to the nucleus [27]. Moreover, it was also observed that last 50 C-terminal aminoacids of nucleoplasmin were able to cross the nuclear pores, accumulating in the nucleus of *Xenopus laevis* oocytes [27]. Taken together, these results suggested that the nuclear import of proteins is a selective process which, in the particular case of nucleoplasmin, is dependent on the presence of a peptide signal present in the carboxylic terminal region of the protein. In spite of these findings, the first nuclear localization signal (NLS) to be identified, at the molecular level, was the NLS of the SV40 large T antigen [28]. The sequence of this NLS was found to consist of seven aminoacids PKKKRKV (Table **1**). This sequence is rich in basic aminoacids, and was demonstrated to be necessary and sufficient to promote the nuclear import of heterologous cytoplasmic proteins, namely β-galactosidase and pyruvate kinase [28]. 

A few years later, Dingwall *et al*. analysed the intracellular distribution of pyruvate kinase fused with C-terminal aminoacid sequences of nucleoplasmin [29]. The authors found that the minimal aminoacid sequence necessary to promote the nuclear import of nucleoplasmin consists of eighteen residues KRPAATKKAGQAKKKKLD. A detailed analysis of this NLS showed that the two domains rich in basic aminoacids are essential to maintain the import activity [30].

The NLSs of the SV40 large T antigen and of nucleoplasmin are now considered as prototypes and are designated as classical or conventional. They consist of a single or bipartite sequence rich in basic aminoacids. Experimental approaches based on the use of fusion protein constructs led subsequently to the identification of a significant number of sequence distinct NLSs (see Table **1**). According to a bioinformatics analysis of the proteome of *S. cerevisiae*, 45% of the proteins annotated in GenBank™ and 57% of the proteins known to be localized in the nucleus, contain a conventional NLS [31]. There are, however, some exceptions. The human c-myc protein contains a NLS constituted by a single stretch of nine aminoacids, PAAKRVKLD, of which only three are basic residues [32]. Besides the NLS of the c-myc protein, there are also a number of already identified NLSs that do not match the classical sequences. 

One the best characterised is the NLS of the hnRNP A1 protein. This NLS, designated M9, is constituted by 38 aminoacids. Surprisingly, this sequence was found to be essential for both the nuclear import and export of the protein [33]. Another example of non-conventional NLSs is represented by the aminoacid sequence responsible for the nuclear import of the ribosomal protein rpL23a. This NLS, also called BIB, consists of a complex 42 aminoacid sequence rich in basic aminoacids [34].

More recently, another structural element bearing properties similar to NLSs was identified in the transcription factor STAT1/STAT1 homodimers and STAT1/STAT2 heterodimers. Nuclear import of STAT1/STAT1 and STAT1/STAT2 dimers is dependent on two elements rich in arginine and lysine residues. These elements are localized in the DNA binding domain of each subunit [35, 36]. The formation of dimers between the STAT transcription factors is catalysed by phosphorylation of a single tyrosine residue in the carboxylic end of the protein by Janus kinases [37]. Janus kinases can be activated by interferon and other extracellular signals. In non-stimulated cells, unphosphorylated STAT transcription factors are predominantly localized in the cytoplasm [38]. However, it was observed that some unphosphorylated STAT proteins can cross the nuclear pore by a mechanism that does require the interaction with transport receptors [38]. In this case, the import is presumably consequence of a direct interaction with the FG motifs of nucleoporins.

There are a number of evidences supporting the hypothesis that some proteins that do not contain an NLS may also be imported to the nucleus [39-42]. These proteins would thus not be able to interact with transport receptors. They seem to be imported through interaction with other proteins that contain a functional NLS. This piggyback mechanism seems to contribute to the nuclear localization of an import defective mutant of the hepatitis D virus antigen [42]. 

## NUCLEAR IMPORT PATHWAYS

The import pathways characterized in more detail involve the participation of the importin-β receptor. Importin-β is thought to be responsible for the nuclear import of all proteins that contain a classical NLS. However, the interaction of importin-β with the aminoacid sequence of the NLS occurs indirectly and involves the participation of other proteins which are members of the importin-α family [43]. The central region of importin-α contains 10 arginine-rich motif (ARM) repetitions in tandem that form a NLS-binding domain [44]. The three-dimensional resolution of importin-α, bound to both monopartite and bipartite NLSs, showed that the central domain contains two specific NLS binding sites [44, 45]. The first binding site, located between the ARM motifs 1-4, directly contacts with the aminoacids of monopartite NLSs and the longer aminoacid sequence of bipartite NLSs. The second binding site of importin-α is located in the ARM motifs 7-8. It binds to the shorter aminoacid sequence of bipartite NLSs [11]. According to data obtained from structural analysis of the protein, mutations in aminoacids of the first NLS binding site severely hamper the interaction of importin-α with both types of classical NLSs [46]. Furthermore, aminoacid substitutions in the second NLS binding site reduce the affinity between importin-α and bipartite NLSs. However, these substitutions do not seem to affect the interaction with monopartite NLSs [46].

Although importin-α directly recognizes the aminoacid sequence of the NLS, the formation of functional import complexes requires the additional interaction between importin-α and importin-β. The interaction between the two importins occurs through the importin beta binding (IBB) domain localized in the N-terminal region of importin-α (Fig. **2-IV**) [47, 48]. Moroianu *et al*. could demonstrate, for the first time that the IBB domain contains a sequence of basic aminoacids similar to a NLS [49]. This sequence has the ability to bind to the central domain of importin-α. Several experimental evidences, based on competition assays, support a model where proteins containing a classical NLS preferentially bind to importin-α in association with importin-β, but with less affinity to free importin-α [50].

The import complexes cross the nuclear pore by a mechanism involving the interaction between importin-β and the FG domains of nucleoporins [17]. Once in the inner face of the NPC, the import complexes are dissociated due the presence of Ran-GTP in the nucleus [50]. As a consequence, the cargos are released and importins are exported back to the cytoplasm (Fig. **2-II**). The dissociation of import complexes starts with the binding of Ran-GTP to three different regions of importin-β [51]. This interaction results in changes of the conformation of importin-β that ultimately hampers the interaction with the IBB domain of importin-α [52]. *In vitro*, the dissociation rate of importin-α/NLS complexes is slow. However, this rate can be accelerated in the presence of exportin CAS bound to Ran-GTP (Fig. **2-IV**) [52]. Exportin CAS is the transport receptor that mediates export of importin-α [53]. 

There are also evidences pointing to a possible participation of nucleoporins in the dissociation of import complexes [52, 54, 55]. Mouse and *S. cerevisiae* Nup2 and Nup50 nucleoporins, respectively, were shown to promote the dissociation of import complexes through direct interaction with importin-α [54, 55]. Structural data and site-directed mutagenesis experiments additionally demonstrated that Nup2 and Nup50 bind to two specific sites in importin-α that are essential for the interaction with exportin CAS bound to Ran-GTP [54]. Upon dissociation of import complexes, importin-β is exported back to the cytoplasm in association with Ran-GTP (Fig. **2-II**). The nuclear import cycle thus ends with the export of importins to the cytoplasm. In this compartment, the GTPase activity of Ran is stimulated by RanGAP and RanBP1 proteins, and after the subsequent GTP hydrolysis, Ran is dissociated from importin-β and exportin CAS (Fig. **2-II**) [53].

All organisms analysed to date were shown to possess a single gene encoding for importin-β [56]. In contrast, the human importin-α family consists of six proteins, encoded by six different genes that are included in three sub-families according to the respective aminoacid sequence homology [43]. With the exception of importin-α6, which can only be detected in testis, all the remaining importin-α proteins are expressed in the majority of tissues analysed [57, 58]. However, the relative expression levels of the different importins-α varies depending on the tissue or cell line [57-59]. All importin-α proteins can efficiently bind to importin-β and exportin CAS. Moreover, they are able to promote the nuclear import of nucleoplasmin and BSA conjugated with the NLS of the large SV40 T antigen in digitonin semi-permeabilized cells [58]. A more recent work, described the effects of inactivation of different importin-α genes in the nuclear import [56]. The obtained results showed that nucleoplasmin is imported to the nucleus independent of the importin-α gene inactivated. This indicates that, at least for some import pathways, different importin-α proteins may play identical roles. In contrast, other proteins like RCC1, seem to preferentially use one single importin-α as receptor to travel to the nucleus [60, 61]. *In vitro* experiments showed that RCC1 binds with higher affinity to importin-α3 [58, 61]. Additionally, silencing of the importin-α3 gene results in inhibition of the nuclear import of RCC1 in about 50% of the analysed cells [56]. These results suggest that the preferential use of an importin-α is determined by the specificity of the interaction with the substrate. However, the aminoacid sequences of the NLS are not sufficient *per se* to determine this specificity [62]. The use of fusion constructs of nucleoplasmin and the NLS of RCC1 showed that this chimeric protein has an increased affinity for importin-α3 when compared with the wild type protein [62]. In contrast, wild type RCC1 fused with the NLS of nucleoplasmin is unable to interact with importin-α3 [62]. Altogether, these results suggest that the three-dimensional conformation of the protein containing the NLS, contributes to promote the specific binding of importin-α to the substrate. 

Besides being responsible for the nuclear import of proteins bearing a classical NLS, importin-β additionally participates in other import pathways that are independent of importin-α. These include the import of UsnRNPs and replication protein A (RPA) [63, 64]. In the case of UsnRNPs, it is thought that nuclear import may involve the direct binding of importin-β or, alternatively, may be mediated by a different from importin-α, adaptor protein [63]. The observation that the nuclear import of UsnRNPs is inhibited in the presence of saturable amounts of analogs of the 5’ RNA cap structure (m7GpppG) and in the presence of UsnRNP central domains, suggests a possible participation of two transport receptors or two transport adaptors [65]. Huber *et al*. found that binding of importin-β to the hypermethylated cap structure of UsnRNAs is mediated by the protein snurportin [66]. Snurportin contains a 40 aminoacid N-terminal domain which is responsible for the direct interaction with importin-β [66]. This domain shares a high degree of homology with the IBB domain of importin-α (Fig. **2-I**) [66]. Similar to importin-α, the basic aminoacid residues of the IBB-like domain of snurportin are essential to promote binding to importin-β [66, 67]. Additionally, the snurportin IBB-like domain contains a second importin-β binding sequence which is homologous to a small region in the nucleoporin Nup153 [68]. Nup153 is localized in the inner face of the NPC, and interacts with several transport receptors, namely with importin-β [69]. Importin-β was found to bind with higher affinity to Nup153 than to snurportin. This affinity difference could account for the dissociation of snurportin and importin-β, in a process similar to the observed for importin-α bound to Nup2/Nup50 [68]. 

The factors that recognize the NLS in the central domain of UsnRNPs and mediate the nuclear import still remain to be identified. However, some evidences point to a possible participation of the survival of motor neurons (SMN) protein complex [70]. One of the roles of the SMN complex is the coordination of the association of Sm proteins to UsnRNAs. This complex remains associated to UsnRNPs upon binding of snurportin to the hypermethylated cap structure of UsnRNAs (Fig. **2-I**) [70, 71]. Interestingly, it was found that the SMN complex directly interacts with importin-β, *in vitro *[70]. The SMN complex remains associated with UsnRNPs during biogenesis of these ribonucleoproteins [71]. This observation, together with the fact that SMN proteins can establish a direct interaction with importin-β, led to the hypothesis that the SMN complex may also participate in nuclear import of UsnRNPs (Fig. **2-I**). In fact, it was possible to demonstrate that the proteins of the SMN complex and importin-β can promote the nuclear import of UsnRNPs in the absence of additional cellular proteins [72]. Moreover, depletion of intracellular SMN complexes hampers the nuclear import of U1snRNP, *in vitro *[72]. Further addition of purified, functional cellular SMN complexes restores the nuclear import of U1snRNP [72]. However, the use of recombinant SMN proteins in the same import assays did not promote the import of U1snRNP thus pointing to a possible participation of additional cellular factors in this process [72]. Supporting this hypothesis, it was possible to demonstrate *in vivo*, that the interaction between importin-β and the SMN complex is indirect and dependent on the presence of RNA [72]. 

Similar to spliceosomal UsnRNPs, the nuclear import of RPA also occurs *via *importin-β and independently of the presence of importin-α [64]. In this case, the adaptor protein is XRP1α [64]. XRP1α binds to importin-β through an arginine-rich basic aminoacid sequence localized in the N-terminal region of the protein. Depletion of RPA results in an almost complete removal of XRP1α from cellular extracts, suggesting that RPA may be the only substrate for XRP1α [64]. 

Importin-β is the only transport receptor of the karyopherin-β family that uses adaptor proteins to interact with the respective substrates. However, as the other members of the karyopherin-β family, importin-β also participates in nuclear import pathways that do not require adaptor proteins to interact with the substrates. One well studied example is represented by the Rex protein of HTLV-1. Rex is imported by a mechanism that involves the direct binding of importin-β to an arginine-rich signal peptide [73]. Histones and ribosomal proteins represent additional examples of proteins containing NLSs that are directly recognized by importin-β, without the participation of additional adaptor proteins [34, 74]. These proteins, however, do not depend exclusively on importin-β to travel to the nucleus [34, 74]. Several proteins of the karyopherin-β family were found to participate in the nuclear transport of both histones and ribosomal proteins. One these proteins is transportin. Transportin participates in the nuclear transport of over 20 proteins, like hnRNP A1 and TAP, involved in mRNA processing [75]. 

In summary, the regulation of nucleocytoplasmic traffic is one of the key steps in controlling gene expression in eukaryotes and several viruses. This traffic occurs through nuclear pore complexes and is regulated by a Ran-GTP gradient. It involves the participation of specialized proteins, named importins or exportins which recognize particular domains in the respective cargos. The recent use of proteomic approaches decisively contributed to draw a detailed picture of the NPC components and structure. Ultimately, this allowed proposing and supporting new models of nuclear import mechanisms. However, these models still need to be tested with further robust experiments before being unequivocally accepted. One of the key features when investigating the active transport through NPCs is the identification of structural motifs in both cargo and receptor proteins. In this context, the advent of genomics provided invaluable tools for the search and identification of transport domains in eukaryotic and virus proteins. In infected cells, several virus proteins are imported to the nucleus where they participate in essential steps of the replication cycle. The detailed identification of mechanisms and signals, both in the cargos and receptors that mediate nuclear import may contribute to identify potential targets for the development of new therapies capable of inhibiting this essential step. 

## Figures and Tables

**Table 1.** Examples of Different Types of Nuclear Localization Signals

NLS Type	Protein	NLS Amino Acid Sequence	Reference
Conventional NLSs	SV40 large T-Ag	PKKKRKV ^132^	[28]
	Polyoma large T-Ag	VSRKRPRP ^196^	[77]
	Hepatitis D virus antigen	EGAPPAKRAR ^75^	[76]
	murine p53	PPQPKKKPLDGE ^322^	[78]
	NF-κB p50	QRKRQK ^372^	[79]
	NF-κB p65	EEKRKR ^286^	[80]
	Human c-myc	PAAKRVKLD ^328 ^/ RQRRNELKRSF ^374^	[32]
Bipartite NLSs	*Xenopus* nucleoplasmin	KRPAATKKAGQAKKKKLD^171 ^	[29]
	Rat glucocorticoid receptor	YRKCLQAGMNLEARKTKKKIKGIQQATA^524^	[81]
	RCC1	MSPKRIAKRRSPPADAIPKSKKVKVSHR ^28^	[82]
Arginine rich NLSs	HTLV-1 Rex protein	MPKTRRRPRRSQRKRPPT ^18^	[73]
	HIV-1 Rev protein	RQARRNRRRRWR ^46^	[84]
Atypical NLSs	Matα2	MNKIPIKDLLNPQ ^13^/ VRILESWFAKNI ^159^	[85]
	Hepatitis B virus core antigen	SKCLGWLWG ^29^	[83]
	Human rpL23a	VHSHKKKKIRTSPTFTTPKTLRLRRQPKYPR-KSAPRRNKLDHY ^74^	[34]
	Human hnRNP A1	NQSSNFGPMKGGNFGGRSSGPYGGGGQ-YFAKPRNQGGY ^305^	[87]
	SREBP2	RSSINDKIIELKDLVMGTDAKMHKSGVLRK-AIDYIKYLQQVNHKLRQENMVLKLANQKNKL^403^	[86]
